# Biomedical Signal and Image Processing for Clinical Decision Support Systems 2014

**DOI:** 10.1155/2015/974592

**Published:** 2015-05-21

**Authors:** Kayvan Najarian, Kevin R. Ward, Shahram Shirani

**Affiliations:** ^1^Department of Computational Medicine and Bioinformatics and Department of Emergency Medicine, Michigan Center for Integrative Research in Critical Care, University of Michigan, Ann Arbor, MI, USA; ^2^Department of Emergency Medicine, Michigan Center for Integrative Research in Critical Care, University of Michigan, Ann Arbor, MI, USA; ^3^Department of Electrical and Computer Engineering, McMaster University, Hamilton, ON, Canada

Abundance of data produced by a growing number of new diagnostic and monitoring devices has provided caregivers with a large number of data streams to consider at the time of clinical decision-making. One of the main challenges of today's medicine is to analyze these tremendous amounts of complex patient data in an increasing number of complex clinical decisions. Moreover, the human eye may not be able to detect and interpret complex patterns hidden in various clinical data, in particular when interpreting physiologic signals and images. Considering the need to make rapid clinical decisions, typically in stressful environments, the urgency to develop efficient quantitative approaches to analyze complex patient data can be further recognized. Specifically, there is clear need for novel signal and image processing algorithms that can create recommendations and/or predictions for healthcare providers, in a variety of clinical decision-making. The performance and capabilities of these quantitative methods are expected to match the complexity and size of the rapidly evolving imaging and measurement systems.

This special issue is the second of the series, intended as an update on the current status of, and advances in, biomedical signal and image processing methods used for clinical decision support systems. The quantitative methods presented in this issue cover a wide spectrum of algorithmic solutions designed for a variety of clinical applications.

The paper by C. Feng et al. introduces an algorithm for correction of lung boundary in X-ray computed tomography (CT) that utilizes split Bregman method and geometric active contour model (ASM). K. B. Kim et al. apply fuzzy ART and image processing techniques to develop an automatic method to extract appendix in ultrasonography. S. Yazdani et al. present an automatic hybrid image segmentation method that integrates the modified statistical expectation maximization (EM) method and the spatial information combined with support vector machines (SVMs) and apply that to segmentation of brain MR images. Modified active contour models (ACMs) are used in the paper by Y. Huang and Z. Liu to segment and track lymphocytes in phase contrast microscopy (PCM) images. In an attempt to improve the endoscopic images, used in diagnosis of various gastrointestinal (GI) tract related diseases, M. S. Imtiaz and K. A. Wahid present a computational method that utilizes an adaptive sigmoid function and space-variant color reproduction for color enhancement. The performances of different methods of feature reduction methods, combined with a variety of classifiers, in detection of malignant tumors in breast images are compared by A. Mert et al. In a paper by M. Sterling et al. a computer-aided clinical decision support system is designed to predict the success of postcardioversion treatments among patients with persistent atrial fibrillation.

As more advanced imaging and monitoring systems are developed and more clinical measurements become available, the quantitative algorithms need to be further improved to analyze the resulting complex data. These algorithms are expected to help not only extract new hidden knowledge from complex patient data, but also provide rapid predictive recommendations to assist healthcare providers in making better and more informed decisions. In addition, it should not be lost that these and other new computational approaches to data will actually better lead us to develop new monitoring and image systems proactively. The papers presented in this special issue outline some of the current computational methods in biomedical and signal analysis.

*Kayvan Najarian* *Kayvan Najarian* *Kevin R. Ward* *Kevin R. Ward* *Shahram Shirani* *Shahram Shirani*

